# The curvilinear relationship between Framingham Steatosis Index and depression: insights from a nationwide study

**DOI:** 10.3389/fpsyt.2024.1510327

**Published:** 2025-01-31

**Authors:** Chunqi Jiang, Bo Wang, Ning Wang, Jun Wang, Yinuo Qu, Guang Zhao, Xin Zhang

**Affiliations:** ^1^ Affiliated Hospital of Shandong University of Traditional Chinese Medicine, Jinan, Shandong, China; ^2^ Pediatrics Department, Central Hospital of Jinan City, Jinan, Shandong, China; ^3^ Basic Medical College, Shandong University of Traditional Chinese Medicine, Jinan, Shandong, China; ^4^ College of Acupuncture - Moxibustion, Shandong University of Traditional Chinese Medicine, Jinan, Shandong, China

**Keywords:** Framingham Steatosis Index, depression, curvilinear, NHANES, cross-sectional study

## Abstract

**Background:**

The Framingham Steatosis Index (FSI) serves as a diagnostic metric for fatty liver. While research has established a link between depression and fatty liver, the association with the Framingham Steatosis Index (FSI) remains undocumented. The aim of this study is to explore the potential correlation between FSI and depression, addressing this research void.

**Methods:**

Our data originates from the National Health and Nutrition Examination Survey (NHANES) database. We employed the PHQ-9 questionnaire for the evaluation of depressive symptoms. We investigated the association between FSI and depression using a weighted multiple logistic regression model and stratified analysis. Non-linear associations were explored using fitted smooth curves. A recursive method was employed to identify inflection points. Subgroup analyses were conducted to examine differences in the association between FSI and depression within subgroups.

**Results:**

Our research encompassed a total of 19,697 participants. Multivariate logistic regression analysis, adjusted for potential confounding factors, demonstrated a significant positive association between FSI and depression, with OR of 1.14 (95% CI: 1.10, 1.18). Stratified analysis indicated that a significant positive correlation exists between FSI and depression among all groups except those with BMI below 30. The non-linear relationship was further confirmed by the restricted cubic splines analysis, which revealed an inflection point at an FSI value of 29.72. Below this threshold, there was no significant correlation, while above it, a positive correlation was observed. Subgroup analysis revealed statistically significant interactions between FSI and depression within the educational attainment groups.

**Conclusion:**

Our study’s discovery is the curvilinear relationship between FSI and depression. Factors such as inflammation, hormonal levels, and metabolic disruptions could be the underlying mechanisms driving this relationship. This finding offers valuable insights that could inform the development of comprehensive intervention strategies for managing depression in clinical settings.

## 1 Introduction

Depression, recognized as a pervasive mood disorder, poses a significant threat to global public health, affecting an estimated 280 million individuals around the world (1) and showing an upward trend in prevalence (2). A cross-sectional study conducted by Neyazi A et al. indicated an alarmingly high prevalence of depression in Afghanistan, reaching 72.05% (3). This condition not only erodes social, psychological, and physical well-being but also amplifies the risk of suicide (4, 5). Individuals with Major Depressive Disorder (MDD) are alarmingly prone to suicidal tendencies, with a mortality rate nearly 20 times higher than that of the broader population (6). In addition, depression is associated with a spectrum of health challenges, such as cardiovascular diseases, breast cancer, sleep disorders, and non-alcoholic fatty liver disease (7–10).

The Framingham Steatosis Index (FSI), developed by Long MT et al. in 2016, is a key diagnostic tool for hepatic steatosis (11). It was established through analysis of a cross-sectional study with 1,181 participants, incorporating factors like age, gender, BMI, triglycerides, hypertension, diabetes, and the ALT: AST ratio. The accuracy of the FSI has been confirmed through validation and it is currently being applied in clinical settings. A cohort study by Nima Motamed and colleagues has demonstrated that FSI is a potent tool for diagnosing non-alcoholic fatty liver disease (NAFLD) and has the additional capability to predict new incidences of NAFLD (12). Furthermore, a community-based evaluation of liver steatosis by Jung TY and other researchers revealed that the FSI outperformed both the Hepatic Steatosis Index (HSI) and the Fatty Liver Index (FLI) in terms of diagnostic accuracy (13). Previous research has already documented associations between liver-related markers and depression. The link between NAFLD and depression could be attributed to the disruption of inflammation, oxidative stress pathways, and mitochondrial dysfunction (14). For example, Manusov EG and colleagues have shown that the AST/ALT ratio is significantly correlated with depression (15). Furthermore, in a cohort study by Cho YK and colleagues, FSI demonstrated a significant predictive ability for cardiovascular risk (16). Considering that cardiovascular diseases, including hypertension, are significantly linked to depression (17), we have grounds to hypothesize a potential relationship between FSI and depression. Nonetheless, the relationship between FSI and depression remains largely unexplored. The National Health and Nutrition Examination Survey (NHANES) constitutes a large, meticulously collected, and extensively detailed database. Utilizing NHANES dataset, this study endeavors to investigate the association between FSI and depression. Our research endeavors to elucidate the intricate interplay among multiple factors and mental health, offering insights to inform the creation of more holistic prevention and intervention strategies.

## 2 Materials and methods

### 2.1 Study participants

**Figure 1:**
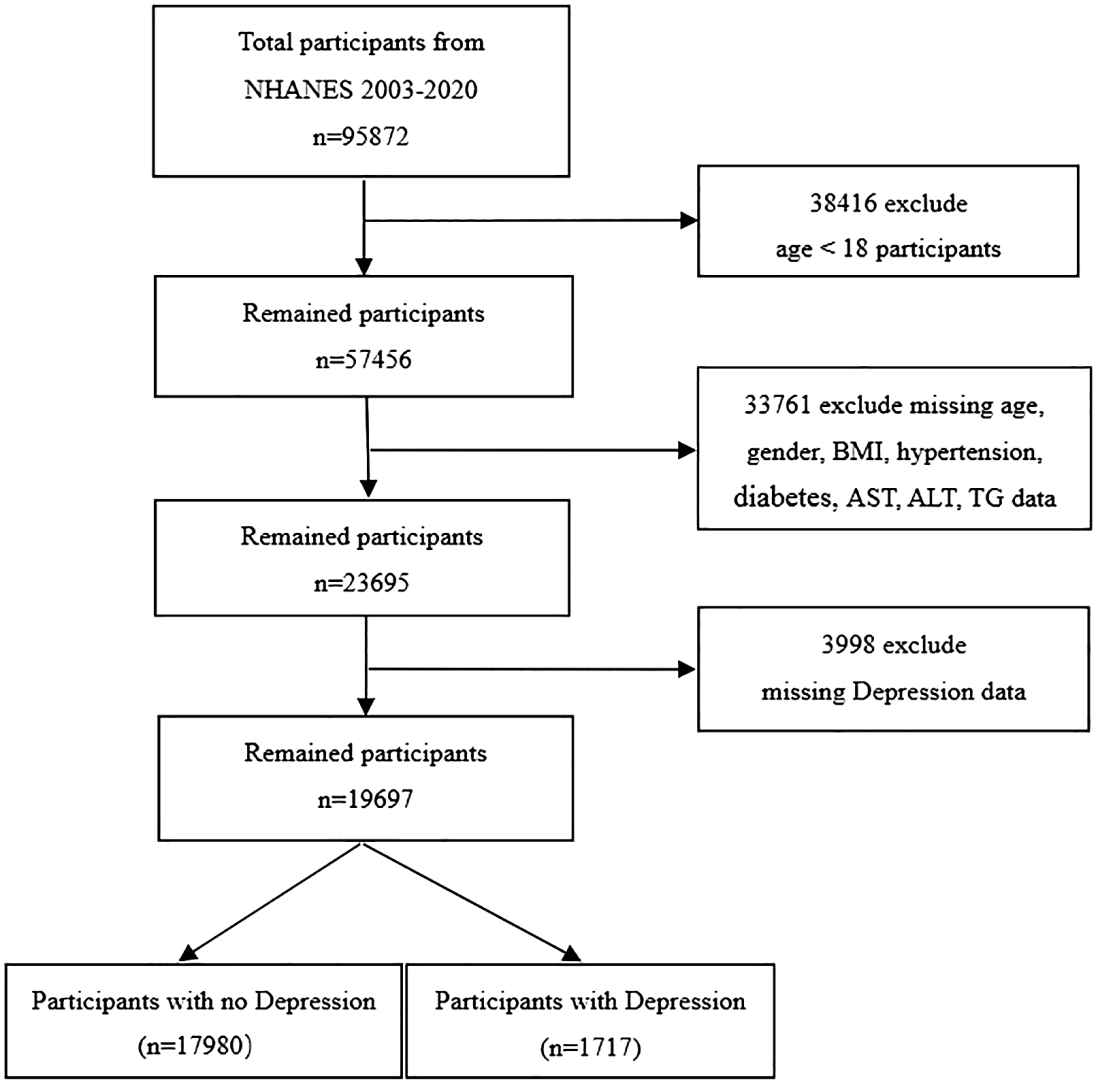
Flow chart of sample selection from the 2003-2020. BMI, body mass index; AST, Aspartate Aminotransferase; ALT, Alanine Aminotransferase; TG, Triglycerides.

Our research drew upon data from the National Health and Nutrition Examination Survey (NHANES) in the United States, spanning nine cycles from 2003 to 2020. The NHANES database offers a rich array of information, including demographic details, lifestyle practices, self-reported health metrics, and blood biochemistry assessments. Data collection is conducted through household interviews, mobile examination centers (MECs), and laboratory tests. This resource is openly available to the research community without the need for specific permissions. The study protocol was granted approval by the National Center for Health Statistics Research Ethics Review Board, and all participants provided their informed written consent. To safeguard the privacy of the individuals involved, all personal identifiers were anonymized. In the data preparation phase of our study, we excluded participants who were under 18 years of age, accounting for 38,416 individuals. Furthermore, we omitted 33,761 individuals due to incomplete data on gender, age, BMI, diabetes status, hypertension, TG, AST, and ALT levels. An additional 3,998 individuals were excluded for lacking depression-related data. Consequently, our study encompassed a total of 19,697 participants, as depicted in **Figure 1**.

### 2.2 Study variables

#### 2.2.1 Definition of depression

The 9-item Patient Health Questionnaire (PHQ-9), recognized as a prevalent self-assessment tool, is fashioned to gauge the intensity of depressive symptoms experienced within a two-week period. This instrument adheres to the criteria for major depressive episodes as detailed in the DSM-IV, the fourth edition of the Diagnostic and Statistical Manual of Mental Disorders (18). This scale is a reliable tool for diagnosing depression, exhibiting high specificity and sensitivity. The questionnaire comprises nine items that evaluate symptoms such as sadness, loss of interest, sleep disturbances, fatigue, feelings of worthlessness, appetite issues, difficulty concentrating, psychomotor agitation or retardation, and suicidal thoughts. Each item is scored from 0 to 3, with 0 indicating “not at all”, 1 reflecting “a few days”, 2 corresponding to “more than half the days”, and 3 signifying “nearly every day”. The total score ranges from 0 to 27, with a score of 10 or above indicating the presence of depression. A total score of 9 or below is considered to indicate no depressive symptoms, while a score of 10 or above is used for the diagnosis of depression, demonstrating 88% sensitivity and 88% specificity. The PHQ-9 exhibits strong internal reliability, as evidenced by a Cronbach’s alpha coefficient of 0.89.

#### 2.2.2 Definition of FSI

We employed FSI, as formulated by Long MT and colleagues in 2016 (11), in our analysis. It is calculated using the formula:

FSI = -7.981 + 0.011 × age (years) - 0.146 × sex (female = 1, male = 0) + 0.173 × BMI (kg/m2) + 0.007× triglycerides (mg/dL) + 0.593× hypertension (yes = 1, no = 0) + 0.789× diabetes (yes = 1, no = 0) + 1.1× ALT: AST ratio>= 1.33(yes = 1, no = 0).

Assessment of TG, AST and ALT Levels: Peripheral blood samples were collected in the morning from participants who had fasted for at least eight hours. Serum alanine TG, ALT levels were assessed using an enzymatic method, while AST levels were measured in serum or plasma by a kinetic rate assay.

#### 2.2.3 Assessment of other variables

CDC collects comprehensive participant data, including demographics, lifestyle, self-reported health status, physical measurements, and biochemical indicators, through computer-assisted personal interviews. Demographic factors encompass age, sex, ethnicity, educational attainment, marital status, and the income-to-poverty ratio. Lifestyle factors, including smoking and drinking habits as well as recreational activities, were also taken into account. Self-reported health data included diabetes, hypertension, stroke, and cardiovascular disease, while anthropometric data included BMI. Biochemical data encompassed gamma-glutamyl transferase (GGT), triglycerides (TG), ALT, and AST. Smoking status was categorized into three distinct groups: “Never” was defined as having smoked fewer than 100 cigarettes in one’s lifetime; “Former” referred to individuals with a history of smoking who had since quit; and “Now” was designated for those who continued to smoke (19). Engagement in recreational activities was binary, recorded as “Yes” or “No.” The diagnosis of diabetes, including pre-diabetes, is based on meeting at least one of the following criteria: 1. Fasting blood glucose levels above 7.0 mmol/L; 2. Hemoglobin A1c (HbA1c) levels of 6.5% or higher; 3. Random blood glucose levels of at least 11.1 mmol/L; 4. Blood glucose levels of 11.1 mmol/L or higher after a 2-hour oral glucose tolerance test (OGTT); 5. A formal diagnosis of diabetes by a healthcare provider; 6. Impaired fasting glucose ranging from 6.11 to 7.0 mmol/L or impaired glucose tolerance, with OGTT levels between 7.7 and 11.1 mmol/L. Hypertension was determined by one or more of the following conditions: 1. Systolic blood pressure readings of 140 mmHg or higher; 2. Diastolic blood pressure of 90 mmHg or higher; 3. Current use of antihypertensive medications; 4. Self-reported hypertension. Alcohol consumption levels were categorized as follows: “Heavy” drinking was characterized by women consuming three or more drinks per day or four or more drinks on a single occasion; men consuming four or more drinks per day or five or more drinks on a single occasion, with at least five heavy drinking days per month. “Moderate” drinking was defined as women consuming two drinks per day and men consuming three drinks per day, with at least two heavy drinking days per month. “Mild” drinking referred to women having one drink per day and men having two drinks per day. “Never” drinking was designated for those who had fewer than 12 drinks in their lifetime, while “Former” drinkers were individuals with a history of drinking who no longer consumed alcohol. CVD was determined through a medical history questionnaire, which recorded whether participants had been diagnosed with coronary artery disease, congestive heart failure, or had a history of a heart attack (20).

### 2.3 Statistical analysis

The research data were properly weighted to accurately represent a more extensive demographic profile. We managed missing data by imputation, utilizing predictive mean matching for continuous variables and logistic regression for binary variables. Participants were categorized into groups with and without depression based on their baseline characteristics. Continuous variables are displayed as the mean ± standard error, while categorical variables are shown as percentages of the overall sample. To explore the association between FSI and depression, we utilized weighted logistic regression analysis. The outcomes are expressed as odds ratios (ORs) along with their respective 95% confidence intervals (95% CI). To substantiate the stability of the association between FSI and depression, we conducted a linear trend analysis. Subsequently, generalized additive models were applied to assess any potential non-linear relationships. After establishing non-linearity, the inflection point was determined using a recursive algorithm, which was then utilized to build a two-piecewise linear regression model. Further subgroup analyses and interaction tests were conducted to identify any additional risk factors that could potentially influence the relationship between FSI and depression. Statistical analyses were performed using R (version 3.5.3) and EmpowerStats software (http://www.empowerstats.com), with a P-value < 0.05 for statistical significance.

## 3 Results

### 3.1 Baseline characteristics

**Table 1** delineates the foundational traits of participants, distinguishing those afflicted with depression from their non-depressed counterparts. Notably, the prevalence of women (63.42%) is markedly higher within the depressed cohort compared to the non-depressed group (49.66%). Individuals with depression are less likely to be married or living with a partner compared to those without depression, and are also less likely to have attained higher education, or to participate in recreational activities. They also have a higher incidence of health issues such as diabetes, hypertension, stroke, and cardiovascular disease. Moreover, they have a lower income-to-poverty ratio and are younger on average, yet they exhibit higher levels of BMI, FSI, TG, and GGT.

**Table 1:** Baseline characteristics of participants.

	No Depression (n=17980)	With Depression (n=1717)	P value
Age (year)	46.87 ± 17.48	46.18 ± 16.41	0.0249
Sex (%)			<0.0001
Female	49.66	63.42	
Male	50.34	36.58	
Race/ethnicity (%)			<0.0001
Mexican American	8.52	8.11	
Non-Hispanic White	67.48	63.29	
Non-Hispanic Black	10.99	13.19	
Other Hispanic	5.51	7.80	
Other Race	7.51	7.61	
Marry status (%)			<0.0001
Never married	18.00	21.74	
Married/Living with partner	64.53	47.82	
Divorced/Widowed/Separated	17.47	30.44	
Education status (%)			<0.0001
Less than high school	4.44	7.94	
High school	34.00	44.61	
More than high school	61.57	47.45	
Recreational activity (%)			<0.0001
No	43.65	65.11	
Yes	56.35	34.89	
Drinking status (%)			<0.0001
Never	11.16	9.31	
Mild	38.01	27.67	
Moderate	18.02	19.09	
Heavy	21.64	27.05	
Former	11.17	16.88	
Smoking status (%)			<0.0001
Never	56.81	39.45	
Now	18.18	38.02	
Former	25.00	22.53	
Diabetes (%)			<0.0001
No	78.58	71.91	
Yes	21.42	28.09	
Hypertension (%)			<0.0001
No	64.27	52.81	
Yes	35.73	47.19	
Stroke (%)			<0.0001
No	97.32	92.54	
Yes	2.68	7.46	
CVD (%)			<0.0001
No	91.76	83.04	
Yes	8.24	16.96	
Income to poverty ratio	3.10 ± 1.63	2.15 ± 1.57	<0.0001
BMI (kg/m2)	28.95 ± 6.85	30.70 ± 8.27	<0.0001
FSI	-1.32 ± 1.74	-0.83 ± 1.98	<0.0001
TG (mmol/L)	1.38 ± 1.16	1.56 ± 1.33	<0.0001
AST (U/L)	24.71 ± 13.53	25.29 ± 22.54	0.0284
ALT (U/L)	24.83 ± 17.54	25.44 ± 29.47	0.0779
GGT (U/L)	27.56 ± 37.52	34.78 ± 54.93	<0.0001

Means ± SE are reported for continuous variables, p value was calculated by the weighted linear regression model. Categorical variables are presented as % with p-values calculated using a weighted chi-square test. BMI, body mass index; CVD, Cardiovascular Disease; AST, Aspartate Aminotransferase; GGT, Gamma-Glutamyl Transferase; ALT, Alanine Aminotransferase; TG, Triglycerides; FSI, Framingham steatosis index.

### 3.2 Association between FSI and depression

**Table 2** details the correlation between FSI and depression. The unadjusted Model 1 revealed a significant positive association, with an OR of 1.17 (95% CI: 1.14, 1.20). This correlation persisted in Model 2, even after accounting for race and education status, with an OR of 1.15 (95% CI: 1.12, 1.18). Model 3, which included all covariates, still showed a positive significant association, with an OR of 1.14 (95% CI: 1.10, 1.18).In the subsequent trend test, the ORs (95% CI) for the association between FSI and depression were Q2 (OR: 0.90, 95% CI: 0.74, 1.09), Q3 (OR: 1.07, 95% CI: 0.89, 1.30), and Q4 (OR: 1.68, 95% CI: 1.41, 2.01), using Q1 as a reference, indicating a potential non-linear relationship between FSI and depression.

**Table 2:** Association of FSI and depression.

Exposure	Model 1 OR (95% CI)	P value	Model 2 OR (95% CI)	P value	Model 3 OR (95% CI)	P value
FSI	1.17 (1.14, 1.20)	<0.0001	1.15 (1.12, 1.18)	<0.0001	1.14 (1.10, 1.18)	<0.0001
FSI quartile						
Q1	Reference		Reference		Reference	
Q2	0.98 (0.83, 1.14)	0.7519	0.93 (0.79, 1.09)	0.3456	0.90 (0.74, 1.09)	0.2906
Q3	1.27 (1.09, 1.47)	0.0019	1.16 (1.00, 1.35)	0.0500	1.07 (0.89, 1.30)	0.4698
Q4	1.92 (1.67, 2.20)	<0.0001	1.76 (1.53, 2.03)	<0.0001	1.68 (1.41, 2.01)	<0.0001
P for trend	<0.0001		<0.0001		<0.0001	
SEX						
Female	1.21 (1.17, 1.25)	<0.0001	1.19 (1.15, 1.23)	<0.0001	1.15 (1.11, 1.21)	<0.0001
Male	1.13 (1.08, 1.18)	<0.0001	1.12 (1.08, 1.17)	<0.0001	1.13 (1.07, 1.19)	<0.0001
Age						
< 60	1.16 (1.13, 1.19)	<0.0001	1.15 (1.12, 1.18)	<0.0001	1.12 (1.08, 1.16)	<0.0001
>= 60	1.21 (1.14, 1.29)	<0.0001	1.20 (1.13, 1.28)	<0.0001	1.19 (1.10, 1.29)	<0.0001
BMI						
<25	1.03 (0.92, 1.15)	0.6227	0.99 (0.88, 1.11)	0.8227	0.86 (0.73, 1.01)	0.0667
25-30	1.08 (1.00, 1.17)	0.0446	1.07 (0.99, 1.17)	0.0991	1.04 (0.95, 1.15)	0.3926
>=30	1.16 (1.11, 1.21)	<0.0001	1.16 (1.11, 1.21)	<0.0001	1.14 (1.08, 1.20)	<0.0001

Model 1: no adjustment.

Model 2: adjusted for race and education status.

Model 3: adjusted for race, education status, smoking status, marriage status, drinking status, physical activity, family income to poverty ratio, CVD, and stroke.

In the subgroup analyses, which are stratified by sex, age, or BMI the model does not incorporate adjustments for the stratification variables themselves. BMI, body mass index; FSI, Framingham steatosis index; OR, odds ratios; CI, confidence intervals.

Sex-stratified analysis reveals that both females and males exhibit significant associations between FSI and depression, with females showing a more pronounced relationship (Female: OR 1.15, 95% CI: 1.11, 1.21; Male: OR 1.13, 95% CI: 1.07, 1.19). BMI-stratified analysis indicates that individuals with BMI >=30 demonstrate a significant association, while those with BMI <30 show no significant link. Age-stratified analysis confirms a consistent association across different age groups, with a stronger association observed in participants younger than 60 years old (<60: OR 1.12, 95% CI: 1.08, 1.16; >=60: OR 1.19, 95% CI: 1.10, 1.29).

**Figure 2:**
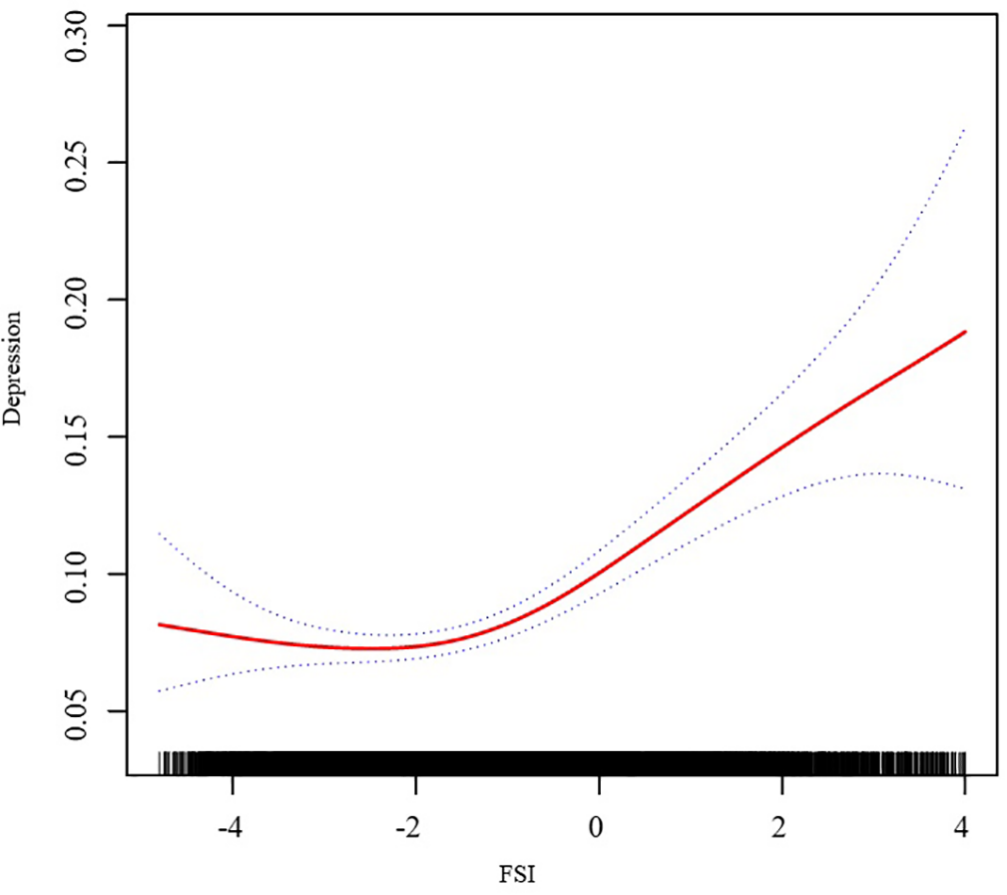
Association of FSI and Depression. We adjusted for race, education status, smoking status, marriage status, drinking status, physical activity, family income to poverty ratio, CVD, and stroke.

In order to further explore the relationship between FSI and depression, we utilized a two-piecewise linear regression model enhanced by RCS analysis. The findings, after accounting for all relevant covariates, exposed a non-linear dynamic between FSI and the incidence of depression, as graphically represented in **Figure 2**. A pivotal point was discerned at an FSI value of -2.4. At values below this threshold, a negative correlation was observed, with OR of 0.89 (95% CI: 0.75, 1.07), which did not achieve statistical significance. Conversely, at values surpassing this threshold, a positive correlation emerged, with an OR of 1.17 (95% CI: 1.13, 1.22), signifying an enhanced probability of depression with an increase in FSI levels, as detailed in **Table 3**.

**Table 3:** Threshold effect analysis of FSI on depression using a two-piecewise linear regression model.

Outcome:	OR (95% CI), P
Fitting by standard linear model	1.14 (1.10, 1.18) <0.0001
Fitting by two-piecewise linear model	
Inflection point	-2.4
< -2.4	0.89 (0.75, 1.07) 0.2127
> -2.4	1.17 (1.13, 1.22) <0.0001
Log-likelihood ratio	0.007

We adjusted for race, education status, smoking status, marriage status, drinking status, physical activity, family income to poverty ratio, CVD, and stroke.

We performed subgroup analyses to investigate the potential Interaction between FSI and the risk of depression among different demographic groups, based on factors such as education level, marital status, smoking and drinking habits. The results indicate that there is a significant interaction between FSI and depression within subgroups defined by education level (**Table 4**). As education levels increase, the association between FSI and depression becomes more pronounced.

**Table 4:** Subgroup analysis.

Subgroup	OR, (95%CI)	P value	P interaction
Drinking status			0.7885
Never	1.10 (1.02, 1.20)	0.0180	
Mild	1.17 (1.10, 1.25)	<0.0001	
Moderate	1.13 (1.04, 1.21)	0.0019	
Heavy	1.13 (1.06, 1.20)	0.0002	
Former	1.16 (1.07, 1.27)	0.0006	
Smoking status			0.3422
Never	1.16 (1.11, 1.22)	<0.0001	
Now	1.10 (1.04, 1.17)	0.0007	
Former	1.15 (1.07, 1.23)	0.0002	
Education status			0.0194
Less than high school	1.06 (0.97, 1.16)	0.1822	
High school	1.10 (1.05, 1.16)	0.0002	
More than high school	1.19 (1.14, 1.25)	<0.0001	
Marriage status			0.9287
Never married	1.13 (1.06, 1.21)	0.0003	
Married/Living with partner	1.14 (1.08, 1.19)	<0.0001	
Divorced/Widowed/Separated	1.15 (1.08, 1.22)	<0.0001	

We adjusted for race, education status, smoking status, marriage status, drinking status, physical activity, family income to poverty ratio, CVD, and stroke but not adjusted for the subgroup analysis variables themselves.

## 4 Discussion

As far as we are aware, this research pioneers the exploration of the association between FSI and the inclination toward depression. The multivariable logistic regression analysis, meticulously adjusted for a spectrum of potential confounding variables, has uncovered a notably positive correlation between FSI and depressive symptoms, with an OR of 1.14 (95% CI: 1.10, 1.18). Our sophisticated curve-fitting analysis has brought to light a nonlinear dynamic between FSI and the presence of depressive disorders, establishing an FSI threshold at -2.4. Below this threshold, no significant link is observed between FSI and the propensity for depression. Above this threshold, however, a 17% heightened risk of depression is associated with each incremental unit increase in FSI. These insights underscore the intricate interrelationship between FSI and the susceptibility to depressive tendencies. The intricate interconnection between physical and mental health is both pervasive and profound. For example, research indicates that an increase of 100μmol/L in uric acid levels is associated with a 21.7% reduction in the risk of depressive symptoms (21). Additionally, studies on the Dietary Inflammatory Index reveal that each unit increase in DII corresponds to a 12% rise in the likelihood of depression (22). These insights not only highlight the significance of the relationship between FSI and depression but also imply that a comprehensive, collaborative approach may be necessary for effective depression management.

Examining the FSI calculation formula reveals that the Framingham Steatosis Index is a composite indicator associated with sex, age, BMI, diabetes, hypertension, ALT/AST, and TG levels. While no research has yet investigated the link between FSI and depression, the relationships between depression and the individual components of the FSI formula have been previously explored in existing studies. Research indicates that women are roughly twice as likely as men to experience depression, a disparity that may stem from differences in sex hormones (23). Among the elderly, a study has found that individuals aged 70 and above who suffer from high blood pressure are at a higher risk of developing depression compared to those aged between 60 and 69 (24). Moreover, it’s been established that an individual’s BMI during adulthood, not during childhood, is causally linked to an increased risk of major depressive disorder (25). This underscores the significant role that age plays in the development of depression. A substantial link has been established between BMI and psychological well-being. Studies have demonstrated that obesity is associated with a 55% increased risk of depression, and individuals with depression are 58% more likely to become obese (26). Additionally, there is a correlation between depression and triglyceride levels; research by Segoviano-Mendoza M et al. has demonstrated that lower cholesterol levels are associated with mood disorders and suicidal behaviors, including Major Depressive Disorder (27). Furthermore, a robust association exists between depression and diabetes, hinting at a potential two-way causality (28). Hypertension, a condition that can severely impact the well-being of older adults, has also been linked to depression (29). There is also evidence to suggest that depression can influence the expression of genetic factors in liver enzymes, particularly the ratio of AST to ALT (30).

The precise mechanisms underlying the interaction between FSI and depression remain unclear. However, evidence suggests that inflammation, hormonal imbalances, and metabolic disruptions may be pivotal in this relationship. Obesity could trigger immune-inflammatory pathways (31), causing adipose tissue to release inflammatory cytokines like tumor necrosis factor-α and interleukin-6, which can impair brain function and precipitate depressive symptoms (32). Moreover, depression might intensify inflammatory responses by affecting the serotonin system and the hypothalamic-pituitary-adrenal axis (33). Research indicates that inflammatory markers such as AST/ALT are significantly involved in the pathophysiology of depression in individuals with diabetes (34). Fluctuations in sex hormone levels could also influence the development of depression by altering immune responses and inflammatory markers like C-reactive protein (35). Depression may further stimulate the hypothalamic axis, leading to increased cortisol secretion and subsequent insulin resistance (36). This insulin resistance could hasten liver fat accumulation and result in the overproduction of very-low-density lipoprotein-triglycerides, causing abnormal triglyceride levels (37). Conditions linked to metabolic disorders, such as hypertension and diabetes, may also contribute to the onset of depression (24). Studies suggest that obesity might elevate the risk of mental health issues through stress responses mediated by the hypothalamic-pituitary-adrenal axis (38). The progression of late-life depression is multifaceted and associated with cognitive decline (24). Educational level also impacts the prevalence of depression, largely due to its influence on memory capacity and, consequently, the expression of depressive symptoms (39). Overall, the interplay between FSI and depression appears to be a complex dynamic involving the dysregulation of multiple physiological systems.

Our data is sourced from the NHANES database, recognized for its stringent and expert-driven data collection, as well as its large sample size, thereby providing robust credibility and reliability to our research findings. Employing stratified and subgroup analyses, we thoroughly investigated the link between FSI and depression, examining its variation across different population subsets. Nonetheless, our study has inherent limitations. As a cross-sectional observational study, it does not establish a causal relationship between FSI and depression. Moreover, unadjusted residual confounders or potential unknown factors may still affect our results. There is currently a gap in research elucidating the relationship and underlying mechanisms between FSI and depression.

Further longitudinal and experimental studies are required to delve into the biological mechanisms in detail. We hope that through our collective efforts, we can improve the understanding of the etiology and pathophysiological mechanisms of depression related to FSI, potentially refining comprehensive prevention and treatment strategies for depression.

## 5 Conclusion

Drawing from the NHANES database spanning 2003 to 2020, our research delineated a non-linear association between FSI and depression, pinpointing a critical inflection point at an FSI value of 29.72, as identified by restricted cubic spline analysis. Below this threshold, no significant correlation was observed, but an affirmative link materialized above it. Stratified analysis consistently showed a positive correlation between FSI and depression across all groups, with the exception of those with BMI below 30. Subgroup analysis further disclosed significant interactions between FSI and depression within educational attainment cohorts, underscoring the influence of demographic factors on this nexus. These findings accentuate the critical role of demographic and clinical parameters, along with tailored management strategies, in the evaluation of depression risk. The observed relationship between FSI and depression implies that interventions such as lifestyle adjustments and specific medical treatments for correlated chronic conditions may be crucial in preventing or alleviating the advancement of depressive symptoms. It is imperative that further clinical and foundational research be conducted to elucidate the mechanisms at play in this association and to devise more effective strategies for the prevention and treatment of depression.

## Data Availability

Publicly available datasets were analyzed in this study. This data can be found here: www.cdc.gov/nchs/nhanes/Default.aspx.
